# Impaired instructive and protective barrier functions of the endothelial cell glycocalyx pericellular matrix is impacted in COVID‐19 disease

**DOI:** 10.1111/jcmm.70033

**Published:** 2024-08-24

**Authors:** M. Smith Margaret, James Melrose

**Affiliations:** ^1^ Raymond Purves Bone and Joint Research Laboratory Kolling Institute, Northern Sydney Local Health District St. Leonards New South Wales Australia; ^2^ Arthropharm Australia Pharmaceuticals Pty Ltd Bondi Junction Sydney New South Wales Australia; ^3^ Graduate School of Biomedical Engineering University of New South Wales Sydney New South Wales Australia; ^4^ Sydney Medical School Northern, The University of Sydney Sydney New South Wales Australia; ^5^ Faculty of Medicine and Health The University of Sydney, Royal North Shore Hospital St. Leonards New South Wales Australia

**Keywords:** blood flow regulation, COVID‐19, endothelial cell, endothelium, glycocalyx, glypican, perlecan, shear flow, smooth muscle cell regulation, syndecan

## Abstract

The aim of this study was to review the roles of endothelial cells in normal tissue function and to show how COVID‐19 disease impacts on endothelial cell properties that lead to much of its associated symptomatology. This places the endothelial cell as a prominent cell type to target therapeutically in the treatment of this disorder. Advances in glycosaminoglycan analytical techniques and functional glycomics have improved glycosaminoglycan mimetics development, providing agents that can more appropriately target various aspects of the behaviour of the endothelial cell in‐situ and have also provided polymers with potential to prevent viral infection. Thus, promising approaches are being developed to combat COVID‐19 disease and the plethora of symptoms this disease produces. Glycosaminoglycan mimetics that improve endothelial glycocalyx boundary functions have promising properties in the prevention of viral infection, improve endothelial cell function and have disease‐modifying potential. Endothelial cell integrity, forming tight junctions in cerebral cell populations in the blood–brain barrier, prevents the exposure of the central nervous system to circulating toxins and harmful chemicals, which may contribute to the troublesome brain fogging phenomena reported in cognitive processing in long COVID disease.

## 1 INTRODUCTION

Human endothelial cells are multifunctional cells that line blood vessels, where they secrete a variety of regulatory bioactive mediators that maintain vascular haemostasis and prevent thrombotic complications. However, when affected by viral infection, stress, hypertension, or alterations in lipid profiles, endothelial cells can become dysfunctional, leading to decreased nitric oxide (NO) expression and alterations in the normal adhesive properties of vessel cells, which result in an elevation in the binding of circulating leukocytes and changes in tight junction adhesion altering vessel permeability.^1^ Accompanying changes in cytokine and chemokine expression profiles can detrimentally effect cellular recruitment resulting in an acute inflammatory response and may lead to chronic inflammation^2^, ^3^ and the development of vascular disease.^4^ Interactions of endothelial cells with dendritic cells, T‐lymphocytes, macrophages and smooth muscle cells generate stimulatory cytokines such as TNF‐α, IL‐1, IL‐6, IFN‐γ and growth factors that support functional and structural vascular changes.^4^ High levels of pro‐inflammatory cytokines can lead to oxidative stress, down‐regulation of endothelial cell nitric oxide synthase (eNOS) activity and elevation in endothelial cell apoptosis. Growth factors such as VEGFA, TGF‐β1 and IL‐8 have chemo‐attractant properties and regulate angiogenic responses in inflammation modulated by NO and eNOS.^3^, ^4^

Endothelial dysfunction resulting in reduced NO levels in blood vessels results in arterial narrowing, resulting in reduced blood flow to the heart, a significant reduction in O_2_ supply to tissues and may lead to angina (chest pain) and cardiovascular complications and even heart attack.^5^ Endothelial dysfunction can occur in diabetes, metabolic syndrome, hypertension, smoking, physical inactivity and COVID‐19 disease.^6^, ^7^, ^8^, ^9^, ^10^, ^11^, ^12^, ^13^, ^14^ Maintenance of a healthy endothelium aids in the control of vasodilation and normalises blood pressure actively suppressing thrombosis, vascular inflammation and hypertrophy.^15^ The glycocalyx covering the luminal surface of vascular endothelial cells consists of a rich network of proteoglycans, glycoproteins and hyaluronan (HA), which collectively regulate vascular permeability^16^ and homeostasis.^5^, ^17^, ^18^ HS‐proteoglycans (syndecans, glypicans and perlecan) uniquely contribute to the ability of the glycocalyx to regulate endothelial permeability. Furthermore, glypican and syndecan on the endothelial cell surface^8^ and perlecan in the pericellular matrix act as shear‐flow biosensors, sending regulatory cues to modulate endothelial cell metabolism. Abnormal shear flow mechanotransductive cues can contribute to the development of atherosclerosis^19^ and can also induce angiogenesis.^20^ Glypican‐1 has been shown to participate in a cooperative mechanism with PECAM‐1 in the mechanotransduction of fluid shear stress to induce NO production, senses flow and phosphorylate platelet/endothelial cell adhesion molecule‐1 (PECAM‐1), leading to eNOS phosphorylation and NO production aiding in vasorelaxation of blood vessels.^21^ Activation of eNOS regulates vascular tone and blood flow by activating soluble guanylate cyclase in the vascular smooth muscle and controls mitochondrial O_2_ consumption by inhibiting cytochrome c oxidase.^22^ Syndecan‐1 and perlecan also act as shear flow biosensors in endothelial cells.^23^, ^24^ Due to its constant exposure to shear flow and circulating neuraminidase, heparanase, hyaluronidase and matrix metalloproteases (MMPs), the endothelial glycocalyx is in a constant state of degradation and regeneration.^25^ A balance in favour of degradation is associated with pathological vascular changes in atherosclerosis, hypertension, vascular ageing, metastatic cancer, diabetic vasculopathies and COVID‐19.^6^, ^7^, ^8^, ^9^, ^10^, ^11^, ^12^, ^13^, ^14^

## 2 PROTECTIVE ROLES OF THE GLYCOCALYX IN HEALTH AND DISEASE

**FIGURE 1:**
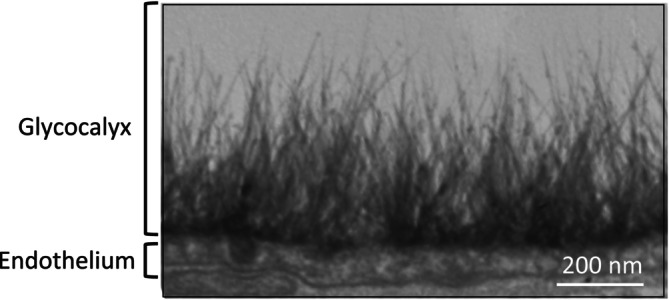
Electron microscope image of a renal artery glycocalyx stained with Alcian blue 8GX showing the frilled appearance of the glycocalyx layer and the underlying endothelium. Image reproduced from Dane et al.^137^

The glycocalyx is a highly hydrated fibrous protein meshwork containing proteoglycans and glycosaminoglycans that surrounds all cells (Figure 1). The thickness of the vascular endothelial cell glycocalyx in humans varies between 0.5 and 5.0 μm depending on vessel type.^26^, ^27^, ^28^, ^29^, ^30^, ^31^ The glycocalyx has a barrier function and has selective permeability properties,^32^ regulates transvascular fluid flow, senses fluid shear forces sending instructive cues to the cytoskeleton of endothelial cells that regulate NO‐mediated vaso‐relaxation to control blood pressure. The glycocalyx also provides anti‐coagulant and anti‐adhesive properties to the surface of endothelial cells preventing thrombus formation on the surfaces of endothelial cells in the lung microvasculature. This can be a problem in the hypercoagulant state of COVID‐19 patients, impairing gaseous exchange through the microcapillaries and causing breathing problems that are often fatal even when these patients are fully ventilated. Alterations in coagulation mediated by the activation of platelets are intrinsically related to viral‐mediated endothelial inflammation.^33^ A further protective effect of the glycocalyx lies in its ability to modulate adhesion of blood leukocytes and platelets to endothelial cells and its ability to shield endothelial cells from oxidative stress.^32^, ^34^, ^35^

## 3 HYALURONAN AND THE GLYCOCALYX OF ENDOTHELIAL CELLS

**FIGURE 2:**
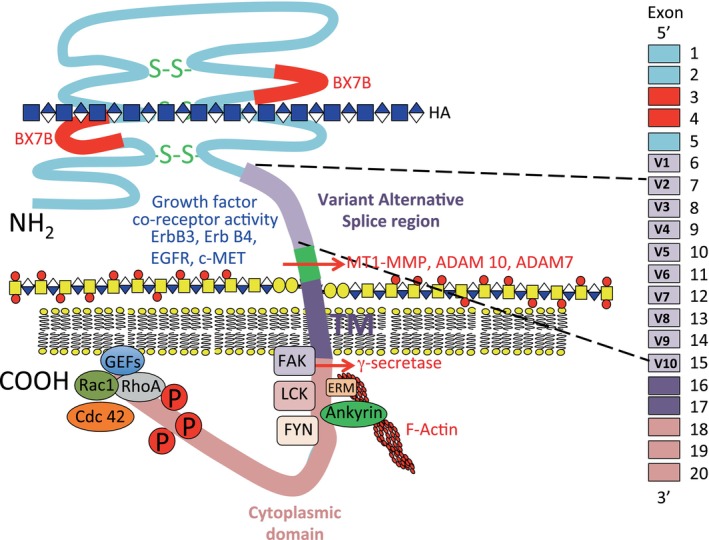
Schematic depiction of the structural organization of CD44 a major endothelial cell HA receptor showing its extracellular disulphide stabilized amino terminal HA binding (BXB7) and alternative splice (V1‐V10) regions, transmembrane (TM) domain and cytoplasmic components including regions of phosphorylation, FAK, LCK, FYN and ERM protein and ankyrin interactive regions. Carboxyl terminal Rac1, Cdc 42, RhoA and GEF interactive regions. Intracellular cleavage sites for γ‐secretase and extracellular cleavage sites for MT1‐MMP and ADAM7 and ADAM10. CD44 has Erb B3, Erb B4, EGFR and c‐MET growth factor co‐receptor activities. FAK, focal adhesion kinase; FYN, tyrosine phospho‐transferase; GEF, guanine nucleotide exchange factor; LCK, lymphocyte specific protein tyrosine kinase; Rac1, Ras‐related C3 botulinum toxin substrate 1.

Hyaluronan is an important protective component of the glycocalyx surrounding all cells. A number of proteoglycans are also important stabilizing functional components of the glycocalyx including the syndecan and glypican family attached to the cell surface and other pericellular proteoglycans such as perlecan, versican and agrin. Proteoglycans interactive with HA (hyalectans) form ternary macro‐aggregate networks that provide a highly hydrated matrix conducive to cellular migration and cellular extension but are also supportive components of the extracellular matrix (ECM) through C‐terminal mediated interactions with matricellular structural glycoproteins, collagen and elastin networks (Figure 2). Perlecan also aids in mechanotransductive osmoregulatory cell‐matrix communication.^36^ Perlecan in the pericellular matrix of blood vessels colocalizes with elastin in the vessel wall providing resilience and visco‐elastic stabilization.^37^ Perlecan‐mediated osmo‐regulatory feedback cues to cells aiding in the regulation of tissue homeostasis.^38^

Extracellular matrix changes in SARS‐CoV‐2‐infected tissues alter their biomechanical and permeability properties and may alter intrinsic mechanosensitive cell signalling pathways that regulate tissue homeostasis and performance. Heparan sulphate proteoglycans are key bioresponsive ECM components that define tissue form and function and are regulated by mechanoresponsive Hippo cell signalling.^39^ A critical role for the evolutionarily conserved Hippo mechanosensory cell signalling pathway has been uncovered in the etiopathogenesis of COVID‐19 disease and represents a potential new mechanistic pathway for the development of this disease.^40^ SARS‐CoV‐2 infection causes activation of the Hippo signalling pathway in the COVID‐19 lung and in in‐vitro cell cultures. Use of Verteporfin, an inhibitor of yes‐associated protein (YAP) activity, which is a transactivator in the Hippo cell signalling pathway, significantly reduces viral replication and represents a novel pharmacological target in the treatment of COVID‐19 disease.^41^

Hyaluronan undergoes depolymerisation during inflammation, high molecular weight HA is anti‐inflammatory but low molecular weight. HA and HA oligosaccharides are pro‐inflammatory and induce MMP synthesis and activation, which may lead to pathologic changes in tissues. Fragmented HA stimulates the expression of inflammatory genes by a variety of immune cells at injury sites and acts as an immune regulator.^42^ HA fragments signal through Toll‐like receptor (TLR) 2, TLR4 and CD44 to stimulate inflammatory genes.^42^ HYBID (HA binding protein involved in HA depolymerisation, KIAA1199/CEMIP) is a key player in HA depolymerisation in skin,^43^ in arthritic synovial fibroblasts and in brain.^44^, ^45^ Depolymerised HA and HA oligosaccharides stimulate the synthesis of MMPs^46^, ^47^, ^48^ which if local TIMP levels are insufficient may result in focal tissue degeneration and generation of free radicals leading to further degradative effects on tissue components.

## 4 ENDOTHELIAL CELL HA RECEPTORS

Endothelial cells express a number of HA receptors. Lymphatic Vessel Endothelial Hyaluronan Receptor 1 (LYVE1) is a type I transmembrane glycoprotein expressed by circulating macrophages and lymphatic endothelial cells.^49^ LYVE‐1 ectodomain is shed from lymphatic endothelial cells by ADAM‐17 and induced by VEGFA and extracellular signal‐regulated kinase (ERK) cell signalling.^49^ Layilin is a novel transmembrane HA receptor containing a C‐type lectin domain that interacts with merlin and radixin^50^ communicating ECM signals to the cell cytoskeleton to modulate cortical structures. Merlin inhibits HA‐CD44 interactions contributing to tumour suppression.^51^ Stabilin 1 and 2 are a family of fasciclin‐like endosomal HA receptors produced by sinusoidal endothelial cells with roles in HA clearance and its fragments from the lymphatic circulation via a network of endothelial sinuses.^52^, ^53^ Clearance of HA fragments from the lymphatics is important since HA fragments can induce MMP synthesis and activation, ECM degradation and inflammation. Stabilin‐1/common lymphatic endothelial and vascular endothelial receptor‐1 (CLEVER‐1) is a multidomain HA receptor of lymphatic and vascular endothelial cells. In adults, stabilin‐1/CLEVER‐1 is a scavenging receptor removing HA from the circulation.^54^ Stabilin‐1 regulates lymphocyte migration within lymphatics and the entrance of leukocytes to sites of inflammation.^55^ HA Receptor for Endocytosis (HARE) is the C‐terminal half isoform of Stabilin‐2 (Stab2/FEEL2) and is generated by proteolysis in‐situ. Stab2/FEEL2 is the full‐length receptor isoform.^52^, ^56^, ^57^ HARE is a multi‐ligand endocytic scavenger receptor clearing HA, CS‐A, C, D, E; heparin, DS, acetylated LDLs, col I and col III N‐propeptides, advanced glycation end products and nucleic acid fragments while Stab2 clears apoptotic, gram negative and positive bacteria and lymphocytes^56^ from the circulatory and lymphatic systems.^58^, ^59^ HARE/Stab2 are transmembrane phagocytic endothelial cell receptors.^60^ Stab2 is transiently expressed at the cell surface but is mainly resident as an intracellular protein.^52^, ^56^, ^57^

## 5 THE SPECIAL CASE OF CD38 AS A MULTIFUNCTIONAL HA BINDING PROTEIN WITH ENZYMATIC ACTIVITY

While CD38 is not a typical HA receptor, it does have useful properties that regulate endothelial cell and monocyte interactions in inflamed tissues. Nicotinamide adenine dinucleotide (NAD) is a cofactor in several oxidation–reduction reactions and regulates cellular energy metabolism and cell signalling. Dysregulation of NAD turnover is associated with many diseases. CD38 is a major NAD‐consuming enzyme with roles in NAD homeostasis and is strongly expressed by endothelial and immune cells.^61^ Elevated CD38 activity is associated with endothelial cell dysfunction and the development of infectious, autoimmune, fibrotic, metabolic, cancer, heart disease and neurodegeneration.^62^ CD38 is a complex ectoenzyme with ADP‐ribosyl cyclase/cyclic ADP‐ribose hydrolase activities, has HA binding properties and is also a leucocyte receptor.^63^, ^64^ Furthermore, IFN‐gamma upregulates monocyte CD38 expression in inflammation, promoting the adhesive properties of monocytes to endothelial cells via interaction with CD31.^65^

## 6 THE GLYCOCALYX AND PULMONARY VASCULAR PERMEABILITY

**FIGURE 3:**
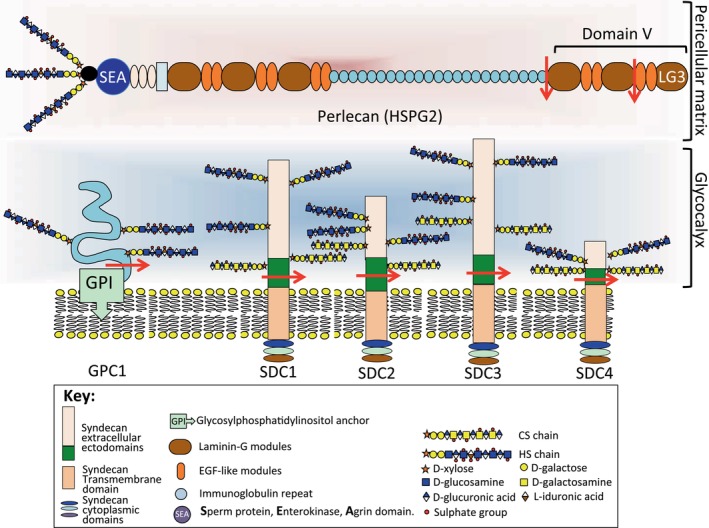
Endothelial cell heparan sulfate proteoglycans, perlecan in the pericellular matrix and glypican‐1 (GPC‐1) and syndecans 1–4 (SDC 1–4) components of the glycocalyx. Sheddase cleavage sites that release GPC and SDC ectodomains are indicated and perlecan cleavages that release perlecan domain V and the LG3 domain of domain V which have therapeutic potential in tissue repair.

The endothelial glycocalyx is a protective layer in the lung endothelium that undergoes dynamic changes in composition due to continual enzymatic degradation, shear‐dependent shedding of its components and reassembly processes.^66^ The healthy endothelial glycocalyx, however, has key roles in the prevention of endothelial dysfunction in pathogenic states, particularly with regard to how it effects vascular permeability and interstitial fluid retention.^66^ Damage to the endothelial glycocalyx is a critical factor in increased pulmonary vascular permeability, which is a basic pathological feature of the severe inflammatory responses induced by the cytokine storm in acute respiratory distress syndrome (ARDS). This results in increased neutrophil adhesion or infiltration and interstitial oedema in lung tissues, and deposition of thrombi on the surface of endothelial cells.^67^ HA and HSPGs (syndecan, glypican families) are major functional components of the lung glycocalyx (Figure 3). The high density of HS in the lung glycocalyx contributes to a dynamic balance in the barrier and permeability properties of this tissue; however, incongruously, HS may also provide an attachment site for invading viruses. In acute lung infection, triggering of an inflammatory immune response results in a rapid increase in lung HA levels and leukocyte infiltration; the water‐regain properties of HA also contribute to interstitial fluid retention in inflamed lung tissues. During inflammation, lower molecular weight HA fragments accumulate in lung tissues; these induce inflammatory chemokine gene expression by macrophages, exacerbating inflammatory conditions.^68^ HA also interacts with a number of hyaladherins and ECM components facilitating assembly of a provisional repair matrix in damaged lung tissue, which eventually leads to a recovery of healthy functional lung tissues. High molecular weight HA also displays interactive properties with immune cells and aids in the maintenance of lung homeostasis, providing healthy functional lung tissues.^69^

## 7 THE GLYCOCALYX AND SARS CoV‐2 INFECTION

Most mammalian cells are between 10 and 100 μm in diameter. The glycocalyx that surrounds these cells has been estimated to have a thickness of 11 μm; however, fixation methodologies required for imaging of the glycocalyx by electron microscopy result in collapse of this structure to some degree. Thus the thickness of the fully hydrated glycocalyx may be considerably larger than 11 μm and therefore would present a significant physical barrier to the movement of viral particles towards the host cell surface.^70^ The SARS‐CoV‐2 virion is an enveloped particle with a diameter of around 60–100 nm.^71^ ACE2 is accepted as the main host cell receptor on host cells. Full‐length angiotensin‐converting enzyme (ACE2) protein is an 805 amino‐acid type‐I transmembrane protein (110–120 kDa) that contains an extracellular PD domain which is the part of the ACE2 molecule that interacts with the Spike glycoprotein of SARS‐CoV‐2. The dimensions of ACE2 dimer imaged by Cryo electron microscopy (EM) is 110 Å × 160 Å. Thus, ACE2 on the cell surface is fully enclosed by the glycocalyx layer.^72^ Neuropilin‐1 (NRP‐1) is a 120 kDa type I transmembrane protein that also acts as a co‐receptor for SARS CoV‐2 interactions and is also fully enclosed by the glycocalyx.

The endothelial glycocalyx contains members of the syndecan and glypican families and perlecan, which orchestrate a myriad of biological processes.^73^, ^74^, ^75^, ^76^ Spatiotemporal regulation of HS structure modulates the functional properties of these proteoglycans with a large range of HS‐binding proteins termed the HS Interactome.^77^ This constitutes a dynamic system precisely regulated in a tissue‐specific and developmentally restricted manner by the variable distribution of the biosynthetic enzymes responsible for HS‐proteoglycan assembly. The biosynthesis of many of these enzymes is regulated by the mechanosensory YAP‐TAZ (transcriptional coactivator with PDZ‐binding motif) Hippo cell signalling pathway.^39^ HS‐proteoglycans in the glycocalyx are highly interactive with components of the complement systems and coagulation cascade. An assessment of the HS Interactome shows many HS binding proteins in the complement and coagulation systems, HS has instructive regulatory properties over many of these proteins and may serve as a global regulator of the complement and coagulation systems.^77^ HS has a proven ability to control complex coordinately interactive protein–protein networks^78^ and is of considerable interest for its potential in therapeutic applications to treat COVID‐19 disease.

Cell surface proteoglycans such as the syndecan family have relatively small core proteins (31–45 kDa) intercalated with the cell membrane; however, these contain relatively large HS side chains (20–60 kDa) projecting away from the cell surface.^79^ Mutual electrostatic repulsion of the GAG components of the HS chains ensures these display an extended conformation providing maximal extracellular coverage. Thus, the HS chains are likely to be the first cellular component that the Spike glycoprotein of SARS CoV‐2 encounters as the viral particles approach prospective host cells. Syndecans have important cell receptor functions and are key regulators of cell signalling and the maintenance of tissue homeostasis.^80^ The endothelial glycocalyx has important protective roles regulating vascular permeability, cell adhesion and has mechanosensory roles over hemodynamic shear stresses and antithrombotic and anti‐inflammatory functions.^78^, ^81^ Inflammatory mediators, reactive oxygen species (ROS) and MMP sheddases that release syndecan ectodomains all contribute to endothelial glycocalyx damage and function in COVID‐19 and are potential areas to target therapeutically.^14^

COVID‐19 disease can be a serious medical condition in the elderly and in patients with pre‐existing health conditions resulting in ARDS.^82^ This is a severe inflammatory condition characterised by a so‐called ‘cytokine‐storm’, which activates inflammatory B and T lymphocytes, natural killer (NK) cells, macrophages, dendritic cells and monocytes.^83^, ^84^ Once activated, these cells produce high levels of inflammatory cytokines such as TNFα and IL‐6 leading to an activation cascade effecting inflammatory cells. TNFα is an inflammatory cell signalling cytokine and a major mediator in acute and chronic inflammation. TNFα is overexpressed in many tissues in COVID‐19‐affected patients with levels correlate with disease severity. Serum IL‐6 levels are also significantly elevated in SARS‐CoV‐2 infected patients with levels that correlating with both viral load and disease progression.^85^ The effective management of this cytokine storm in ARDS is a major challenge in the treatment of COVID‐19 infections.^86^, ^87^

Haemolysis is a common feature of COVID‐19‐infected tissues.^88^, ^89^, ^90^, ^91^ This leads to further detrimental effects on these tissues with free heme resulting in oxidative stress, local generation of NO oxygen free radicals and mitochondrial distress, leukocyte recruitment, vascular permeabilization, platelet and complement activation, thrombosis and fibrosis leading to impaired tissue function particularly in the lung and liver.^92^, ^93^ Platelets initiate blood clotting, severely affected COVID‐19 patients display a high incidence of hypercoagulation (excessive blood clotting) in the lungs and brain.^94^, ^95^, ^96^, ^97^ Fibrinogen levels are elevated in severely affected COVID‐19 patients.^98^, ^99^ Fibrinogen has major roles in the initial stages of formation of fibrin clots that bind platelets and plasma proteins to form a haemostatic plug in coagulation and wound closure. In pathological coagulative states this network entraps large numbers of erythrocytes and leukocytes forming a thrombus in blood vessels, elevated fibrinogen levels in COVID‐19 disease promotes hypercoagulation.^100^ D‐Dimers are generated by the action of plasmin on cross‐linked fibrin and are indicative of coagulation and fibrinolytic system activation.^101^ D‐Dimer levels are predictive of the clinical severity of COVID‐19. Escalated anticoagulation based on D‐dimer levels predicts a lower risk of mortality in severe COVID‐19 patients.

COVID‐19 patients who develop a severe proinflammatory state are also frequently associated with a procoagulant endothelial phenotype.^102^ COVID‐19 infection produces an elevation in fibrinogen and D‐dimer/fibrin(ogen) degradation products associated with systemic hypercoagulability.^103^ D‐dimer levels positively correlate with mortality rates in COVID‐19 patients and lead to arterial thrombotic events including strokes, ischemic limbs and microvascular thrombotic disorders in the pulmonary vascular beds.^104^ While COVID‐19 patients have low platelet levels and mild thrombocytopenia, they also have increased platelet consumption and a corresponding increase in platelet production. Megakaryocytes are major producers of platelets. HS is a critical regulator of the immunoreceptor tyrosine‐based inhibition motif (ITIM)‐containing receptor G6b‐B‐R that regulates platelet production and activation.^105^ Binding of G6b‐B‐R to the HS side chains of perlecan and multivalent heparin inhibits platelet and megakaryocyte function by inducing downstream signalling via the tyrosine phosphatases Shp1 and Shp2. SARS‐CoV‐2 initiates programmed cell death in platelets^106^; thus, G6b‐B‐R has important roles to play maintaining platelet levels to participate in wound healing responses.^107^ The interaction of perlecan with G6b and G6b‐R regulates fibrotic changes in tissues produced by excessive levels of platelet activation.^107^ Perlecan HS also regulates cell adhesion, proliferation and growth factor signalling in tissue repair responses and maintains tissue homeostasis and optimal tissue function.^108^, ^109^

In hypercoagulation, the fibrinolytic system attempts to re‐balance the coagulation system; however, in COVID‐19, this can be overwhelmed by the hypercoagulative state that prevails.^110^ Plasmin is a major clot‐dissolving fibrinolytic enzyme and is produced with elevated levels of tissue plasminogen activator (tPA), which in turn is regulated by plasminogen activator inhibitors‐1 and ‐2 (PAI‐1, PAI‐2).^111^ Autopsies of COVID‐19 fatalities have revealed that thrombosis, micro‐angiopathy, haemorrhage and alveolar damage are common features of COVID‐19‐affected lung tissues.^112^ Furthermore, the dyslipidemia displayed by COVID‐19 patients results in abnormally high levels of low‐density lipoproteins (LDLs) and low levels of high‐density lipoproteins (HDLs).^113^

## 8 THE SELECTINS AND LEUCOCYTE TRAFFICKING IN INFLAMMATION

Selectins are a family of variably glycosylated closely related cell surface sialo‐glycoproteins ranging in size from 74 to 240 kDa in size. p‐selectin is expressed on platelets and leucocytes, e‐selectin on endothelial cells and l‐selectin on leucocytes monocytes, neutrophils and eisinophils.^114^, ^115^ Selectins contain an N‐terminal Ca^2+^‐dependent lectin domain, EGF domain and variable numbers of short repeat peptides homologous to complement binding sequences, a transmembrane domain and a short cytoplasmic tail. Selectins have roles in leucocyte trafficking, early stages of leucocyte extravasation and rolling of leucocytes over the surface of endothelial cells, integrin activation and ROS production by leucocytes. (X5, X6ref) Ligands for the selectins include Sialyl Lewis‐X oligosaccharides, CD24 and p‐Selectin glycoprotein ligand‐1 (PSGL‐1). PSGL‐1 expressed on leucocytes binds to p‐selectin in the vascular endothelium and l‐selectin on neutrophils to promote rolling; e‐selectin has roles in leucocyte recruitment in inflammation.^116^

Coronary artery disease and associated complications are leading causes of death worldwide. Inflammatory activation and dysfunction of the endothelium are key events in the development and pathophysiology of atherosclerosis and are associated with an elevated risk of cardiovascular events.^117^ A greater understanding of the pathophysiologic mechanisms underlying endothelial dysfunction and atherosclerosis progression and identification of biomarkers and therapeutic strategies to prevent endothelial dysfunction and will reduce the risk of development of coronary heart disease. Furthermore, GPC1 modulates FGF, VEGFA, TGF‐β, Wnt, Hedgehog and BMP‐mediated cell signalling and is a potential therapeutic target in cancer.^118^

## 9 ENDOTHELIAL CELL‐COMPLEMENT SYSTEM CROSSTALK IN COVID‐19 DISEASE

The complement system is an arm of the immune system that acts along with antibodies and phagocytic cells of the immune system to combat microbial infection.

While viral protection afforded by Complement is effective against many viruses, it does not seem to inactivate SARS CoV‐2 and may actually contribute to endothelial cell dysfunction that leads to some of the detrimental symptomatology of COVID‐19 disease.^119^ COVID‐19 is associated with an unusual prothrombotic state, intense endothelial activation and vascular changes, activation of platelets and upregulation of the coagulation cascade plus impaired fibrinolysis, which all contribute to a hypercoagulable state characteristic of COVID‐19.^120^

In a recent study by Cervia‐Hasler et al.^121^ multimodal proteomics of COVID‐19 patient serum samples over a 12‐month period confirmed severe SARS CoV‐2 infection, terminal Complement system dysregulation and ongoing activation of the alternative and classical complement pathways. Biomarkers of haemolysis, tissue injury, platelet activation were all increased with Complement and thromboinflammatory biomarkers most prominent suggesting that these may be useful in diagnostics. Ruf^122^ also showed that during acute SARS CoV‐2 infection, activation of the complement and coagulation systems occurred in Long Covid patients. Damage to endothelial cells through membrane insertion of the complement C5b‐C7 complex was proposed to result in the release of prothrombotic multimeric von Willebrand factor and thrombospondin‐1 from the endothelium. vWF multimers recruit platelets and promote thrombin generation, while TSP1 promotes monocyte‐platelet interactions that also promote thrombus formation. Moreover, reduced levels of ADAMTS13 in Long Covid patients and the accumulation large vWF multimers induces C3b binding and the activation of the alternative complement pathway, which along with activation of the coagulation system results in microclot generation that may explain some of the symptomatic features of Long Covid Disease.

## 10 ENDOTHELIAL CELL THERAPEUTICS AND GAG MIMETICS

The endothelium should not be considered a passive victim of inflammatory processes in atherosclerosis, diabetes, coronary artery disease and acute myocardial infarction. Rather, it should be considered as an active player; a search for therapeutic compounds that modulate endothelial cell activity using modern methodology and equipment will undoubtably yield beneficial results in the aforementioned conditions.^1^ GAG mimetics is a powerful therapeutic strategy to modulate endothelial cell activity. Activated endothelial cells and platelets express p‐selectin, a high kinetic adhesion receptor required for leukocyte rolling in the inflammatory response and in tumour cell binding during metastatic events. Inhibition of the adhesive properties of p‐selectin is a promising therapeutic approach in the treatment of these conditions. Heparin has anti‐inflammatory and anti‐metastatic therapeutic activities due to its ability to inhibit the binding properties of p‐selectin and e‐selectin. Pentosan polysulphate (PPS), a semi‐synthetic heparinoid sulphated xylan shows significant potential targeting specific cell populations in therapeutic tissue repair applications.^123^ PPS stimulates HA production by a number of cell types maintaining the quality of the endothelial glycocalyx.^124^ PPS also regulates growth factor and cytokine production to preserve tissue homeostasis and equips PPS with tissue and cell protective properties which show significant potential in the modification of disease processes.^124^, ^125^, ^126^, ^127^

## 11 CONCLUDING REMARKS

It is well established that the glycocalyx has important cell protective properties.^128^ HS in particular has critical protective roles to play in the pulmonary endothelial cell glycocalyx.^129^ Heparan sulphate (HS) proteoglycans (HSPGs) are the most abundant GAGs in the endothelial glycocalyx and constitute 50%–90% of the total proteoglycans in the endothelial glycocalyx.^130^ The sheddase activity of MMPs and detrimental cellular effects mediated by the cytokine storm of ARDS impact on the endothelial cell glycocalyx in COVID‐19 disease and reduce its protective properties.^82^ Some studies have addressed the question of whether the glycocalyx might be replenished or its functional properties improved in disease.^131^, ^132^, ^133^ Analysis of the HS atherosclerosis interactome has been used to develop more effective function‐defining HS isoforms and HS interactive partners.^134^ The vaso‐dilatory anaesthetic sevoflurane has been reported to promote regeneration of the endothelial glycocalyx by upregulating sialyltransferase activity.^135^ Advances in functional glycomics^136^ thus suggest that repair of the damaged glycocalyx may be possible.

## 12 CONCLUSIONS

The endothelial cell is a key component of many tissues and is impacted by COVID‐19 disease, resulting in many of the symptoms associated with this viral disorder. Pharmaceutically targeting the endothelial cell is a logical approach in the treatment of this condition. Improvements in GAG mimetic research holds promise as a treatment option against SARS‐CoV‐2, both in a preventative capacity to avoid initial infection and in a reparative capacity for the treatment of much of the associated COVID‐19 symptomatology.

## AUTHOR CONTRIBUTIONS

**M. Smith Margaret:** Conceptualization (equal); formal analysis (equal); writing – original draft (equal); writing – review and editing (equal). **James Melrose:** Conceptualization (equal); formal analysis (equal); funding acquisition (equal); project administration (lead); writing – original draft (lead); writing – review and editing (lead).

## FUNDING INFORMATION

This study was funded by the Melrose Personal Research Fund, Sydney, Australia.

## CONFLICT OF INTEREST STATEMENT

J.M. has received consultancy fees from Arthropharm Pharmaceuticals Pty Ltd, Bondi, Sydney, Australia. M.M.S. is a clinical research director at Arthropharm Pharmaceuticals Pty Ltd. The authors have no conflicts to report. This company had no input into the interpretation of this study or the reason to publish.

## Data Availability

All data is available from the individual studies cited.
